# Building healthcare provider relationships for patient-centered care: A qualitative study of the experiences of people receiving injectable opioid agonist treatment

**DOI:** 10.1186/s13011-020-0253-y

**Published:** 2020-01-20

**Authors:** Kirsten Marchand, Julie Foreman, Scott MacDonald, Scott Harrison, Martin T. Schechter, Eugenia Oviedo-Joekes

**Affiliations:** 1 School of Population and Public Health, University of British Columbia, 2206 East Mall, Vancouver, BC V6T 1Z3 Canada; 2 Centre for Health Evaluation & Outcome Sciences, Providence Health Care, St. Paul’s Hospital, 575- 1081 Burrard St, Vancouver, BC V6Z 1Y6 Canada; 3 Providence Health Care, Providence Crosstown Clinic, 84 West Hastings St, Vancouver, BC V6B 1G6 Canada

**Keywords:** Patient-centered care, Injectable opioid agonist treatment, Opioid use disorder, Patient-reported outcomes, Grounded theory

## Abstract

**Background:**

Injectable opioid agonist treatment (iOAT) was designed as a pragmatic and compassionate approach for people who have not benefitted from medication assisted treatment with oral opioids (e.g., methadone). While, a substantial body of clinical trial evidence has demonstrated the safety and effectiveness of iOAT, considerably less is known about the patient-centered aspects of this treatment and their role in self-reported treatment goals and outcomes. The aim of this study was to explore participants’ experiences in iOAT as they broadly relate to the domains of patient-centered care. A secondary goal was to explore how these experiences affected participants’ self-reported treatment outcomes.

**Methods:**

A qualitative methodology, and constructivist grounded theory approach, was used to guide sampling, data collection and analysis. A total of 30 in-depth interviews were conducted with people receiving iOAT in North America’s first clinic. Audio-recordings for each semi-structured interview were transcribed and read repeatedly. The strategy of constant comparison was used through iterative stages of line-by-line, focused and theoretical coding until theoretical saturation was achieved.

**Results:**

*“Building healthcare provider relationships for patient-centered care in iOAT”* was the emergent core concept. Healthcare provider relationships were established through two interrelated processes: ‘Opening up’ was attributed to the positive environment, and to feeling understood and supported by healthcare providers. ‘Being a part of care’ emerged as participants felt safe to ask for what was needed and had opportunities to collaborate in treatment decisions. These processes established a foundation in which participants experienced care that was responsive to their individual dose, health and psychosocial needs.

**Conclusions:**

The core concept suggested that therapeutic relationships were fundamental to experiences of patient-centered care in iOAT. When relationships were respectful and understanding, participants received individualized and holistic care in iOAT. These findings offer a valuable example of how therapeutic relationships can be strengthened in other substance use treatment settings, particularly when responding to the diverse treatment needs of clients.

## Background

The increasing prevalence of illicit opioid use is a major global concern due its severe health consequences [1]. Its dramatic harms are most apparent in North America where opioid-related overdoses are a leading cause of preventable deaths [1, 2]. This ongoing crisis urgently calls for a diversification of medication-assisted treatments (MAT) [3, 4].

MAT with oral buprenorphine or methadone remains the mainstream clinical treatment in many countries [1]. In addition, some European countries [5], and very recently Canada [6, 7], deliver injectable opioid agonist treatment (iOAT). Under this approach, people who have not improved with OST are provided with injectable diacetylmorphine or hydromorphone. These medications are taken daily in clinical settings and under the observation of healthcare staff [5, 8, 9].

Evidence for the safety, effectiveness and cost-effectiveness of iOAT has been established through several randomized controlled trials [10–17]. In addition, qualitative sub-studies have provided initial descriptive data about participants’ experiences with iOAT during the trial period [18–21]. These studies revealed participants’ appreciation for care that was individualized, holistic, respectful, and honest [18, 19]. Participants also described experiencing outcomes that were beyond those typically measured in the clinical trials, including positive changes to daily routines and improved self-esteem [18, 19].

Themes emerging from these earlier studies allude to experiences of patient-centered care (PCC), which promotes a personalized, holistic, empowering and respectful approach [22–28]. Despite increasing interest in PCC for substance use treatment [29–33], few studies have examined this approach in the treatment of opioid use disorder [34]. Set in Canada’s first iOAT clinic, this qualitative study explored participant’s experiences in iOAT and self-reported outcomes, as they broadly relate to PCC. It addresses two important gaps. First, it deepens understanding of the patient-centered attributes of iOAT. Second, it demonstrates the interrelationship between principles of PCC and builds upon existing conceptualizations of PCC [32].

## Material and methods

### Design, setting and participants

This qualitative study followed a constructivist grounded theory approach [35, 36], selected for its ability to understand how and why patient-centered experiences were relevant in iOAT. In-depth interviews were conducted with clients receiving iOAT at Providence Health Care’s Crosstown Clinic (Vancouver, Canada). This Clinic was initially implemented as the purpose-built site for the NAOMI (2005–2008) [17] and SALOME (2011–2014) [16] clinical trials that tested the effectiveness of injectable diacetylmorphine and hydromorphone treatments. At the time of collecting data for the present study, Crosstown Clinic remained the first and only iOAT program in North America, delivering treatment to approximately 130 people with opioid use disorder [16, 37]. In this setting, clients are prescribed up to three doses per day and self-administer medications under the observation of Registered Nurses [38]. During the present study, Physicians were the primary prescribers of iOAT and participants had access to an interdisciplinary care team of Health Professionals (Social Workers, Psychiatrist, Nurse Practitioner, a Nutritionist).

Consistent with the grounded theory approach, purposeful and theoretical sampling of Crosstown Clinic clients evolved iteratively with data analysis and continued until reaching theoretical sensitivity (i.e., no remaining questions about the interrelationship between core concepts) [36]. This occurred after conducting 30 in-depth interviews with 14 women and 16 men (Table 1).

**Table 1:** Select self-report participant characteristics at initial iOAT entry

Characteristics	*N* = 30
	M ± SD; n (%)
Socio-demographic characteristics	
Age	44.7 ± 8.7
Women	14 (46.7)
Any Indigenous vs. Non-Indigenous ancestry ^a^	7 (23.3)
Any non-stable housing in prior 3 years vs. none	19 (63.3)
Any street housing in prior 3 years vs. none	6 (20.00)
Education	
Less than high-school certificate	10 (33.3)
High school certificate	7 (23.3)
High school certificate and higher (e.g., trades, university)	13 (43.3)
Health status	
Physical health score ^b^	13.8 ± 8.0
Psychological health score ^b^	9.5 ± 8.0
Health related quality of life score ^c^	0.8 ± 0.2
Susbstance use and substance use treatment history	
Lifetime years heroin injection	14.5 ± 8.6
Times ever attempted medication assisted treatment with oral methadone	4.2 ± 2.6
Highest daily dose of oral methadone in mgs ^d^	106.7 ± 51.9
Ever enrolled in outpatient withdrawal	28 (93.3)
Ever in outpatient counseling	23 (76.7)
Ever enrolled in residential treatment	17 (56.7)

Data shown are mean ± standard deviation; N (%)

^a^Aboriginal ancestry includes participants who self-identified as Inuit, Metis, or First Nations.

^b^MAP Physical and Psychological health scores range from 0 to 40 with higher scores indicating poorer health.

^c^EQ5D (Euroquol) with Canadian weights scores range from 0 to 1; higher scores are indicative of better health status.

^d^Based on administrative prescription records data from 1995 to 2012.

### Data collection

All interviews took place in a private research office that was independent of the clinical site. In-depth interviews (conducted by author KM) used open-ended questions to capture participants’ experiences in iOAT as they broadly related to the four core domains of PCC: (1) individualized care tailored to clients’ unique needs, values and preferences; (2) a holistic or bio-psycho-social perspective; (3) an enhanced therapeutic relationship; and (4) client empowerment and participation in treatment decisions [22–28, 32]. Interviews lasted an average of 48 min (range 18–91 min) and participants received a $20 honourarium for their time. Interviews were audio-recorded in order to maintain close attention to the unfolding conversation. Audio-recordings were transcribed verbatim, read repeatedly and underwent initial coding immediately after the interview.

### Analysis

The core principles of grounded theory were followed, including simultaneous data collection and analysis, a blended inductive and deductive approach to analysis, the strategy of constant comparison, the use of memos and sampling for theoretical development and sensitivity [36]. Initial coding was done by in-vivo (directly quoted terms, e.g., “constant grind”) and line-by-line codes (labeling each line of transcript) to become immersed in the language, symbols and actions used by participants. Focused coding was subsequently used to uncover categories that were most significant and synthesized the data across transcripts. Comparisons were continually made within and between these emerging categories as new data was collected, transcribed and coded.

Analysis of early interviews pointed to interactions with healthcare providers as a core category. Therefore, ongoing sampling and data collection focused on understanding this category (e.g., for whom does this matter, under what circumstances) and its relationship with other domains of PCC (e.g., “how does mutual trust impact your medication dose?”). This was achieved through theoretical memoing, diagramming, re-examining the collected data and studying extant texts [36].

## Results

**Fig. 1:**
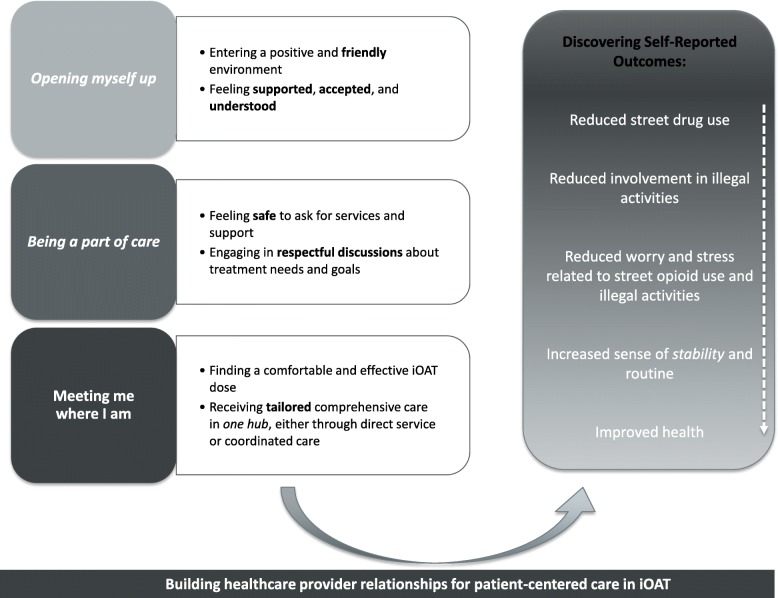
Building healthcare provider relationships for patient-centered care in iOAT. Panel on left displays the categories and sub-categories that defined the core concept. *“Building healthcare provider relationships for patient-centered care in iOAT”.* Categories were: *Opening up*, *Being a part of care*, and Meeting me where I am. Italicized text reflects in-vivo quotes from participants. Panel on right defines the categories that emerged through the second core concept, *“Discovering self-reported outcomes”*

Figure 1 depicts the core concepts that emerged: ‘*Building healthcare provider relationships for patient-centered care in iOAT’* and ‘*Discovering self-reported outcomes’*. The main categories of these concepts and their interrelationship are detailed below.

### Building healthcare provider relationships for patient-centered care in iOAT

This core concept was composed of three interrelated categories: opening myself up; being a part of care; and meeting me where I am (Fig. 1). “*Opening myself up”* (N29) was a process that began with the clinic environment itself and with daily interactions that were positive and *“friendly”.* Participants expressed that healthcare providers used these daily interactions to “*go out of their way to make sure that you’re okay,* [and] *that things are well in your life”* (N23)*.* The ongoing experience of positive interactions encouraged participants to *“let your guard down…*[and] *just be yourself”* (N25).

Seeking clarification regarding why participants needed to “let their guard down” revealed the perception that *“heroin addiction is a private thing…a lot of us have huge trust issues…so it takes time to get to know somebody, to trust them to be around you while you’re doing this private thing”* (N12). These “trust issues” were also rooted in participants’ prior experiences of discrimination in the healthcare system related to their use of street opioids.

*“*[My iOAT doctor] *treats people with respect and dignity, which is huge.* [For] *many years in my life, I’ve been ‘the junkie’… Looked down on by people in that* [healthcare] *field, no matter where or what their position was. I don’t get that there* [at Crosstown Clinic]*… The staff is amazing... I really feel like each and every one of them cares.”* (N10)

As participants’ trust in healthcare providers grew, they discovered that staff also had *“a lot of understanding…. A lot more understanding than the average person”* (N24). Understanding and respectful care was a premise of the second category *being a part of care*. There were two salient properties of this experience. The first was a sense of *“safety”* to speak up about iOAT needs and preferences. For example, as one woman explained:

*“You have to remember, it’s not like going to see another medical doctor, it’s okay to say that you’re still not well, or to let them know what you need or don’t need. They’re very open minded to that...”* (N17)

The second theme demonstrated that *being a part of care* required opportunities for participants to express their opinions and to have *“input into what we need…rather than being told what we need”* (N01). As this participant explained:

*“Unless it’s going to harm me and my health somehow*, [my doctor will] *listen to me.* [My doctor] *knows from my past with working with* [her/him] *that I’m not going to take advantage. There is no taking advantage there. Really like that whole sort of shame-based way of thinking of things isn’t there, so you really feel safe asking for what you need. I’ve gone down on my dose because I felt like that’s what I need, and then realized it was wrong and then gone back up. And it’s really up to me there.”* (N01)

Learning that healthcare provider’s open-minded and respectful communication style was fundamental to shared decision-making prompted new insights into the process of PCC. It suggested that a positive therapeutic relationship was fundamental to experiencing care that was *meeting me where I am.* As shown in Fig. 1, this category was defined by two processes: finding a comfortable and effective dose and receiving tailored comprehensive care.

Exploring the first of these experiences revealed that effective and comfortable doses were person-specific. For example, some participants described higher doses as being more effective to reduce street heroin use because of their *“high tolerance to opiates”* (N25) or *“so, I didn’t feel the street heroin, and eventually I just stopped wasting my money”* (N22). Other participants suggested that their preferences changed over time. Initially they sought higher doses and gradually lowered them until finding *“a comfortable dose, where it’s enough to get me through the whole day and I sleep good at night”* (N19).

Participants also preferred an individualized approach to treatment planning because their expectations for the duration of iOAT were also unique. At the time of the interviews, many participants planned to remain on iOAT *“for as long as I need it”* (N09); some were beginning to think that it was *“time to move on”* (N26) from medication-assisted treatment altogether; and few were in the midst of planning their transition. As these and later quotes (see section 3.2) suggested, the duration of treatment “*depends on the person”* (N09), their goals, and the complexity of their opioid dependence history. As one participant explained, *“those behaviors had become so engrained after such a long period of time that a month or two in a treatment center is really nothing”* (N23).

Related to these “engrained behaviours”, holistic care was defined by participants as a core feature of iOAT; *“it’s the doctor with your meds, it’s the social workers, it’s the dietitian…that’s all part of my well-being and my survival day to day”* (N17). Participants also appreciated that support was available *“all in one hub”,* either through direct service or coordinated care. Beyond this being more convenient, centralized care was closely aligned with participants’ needs. As one participant explained, “*when you are trying to get some order in their life…you can’t have* [us] *going all over the place and that, to have it all in one hub, it’s right there man”* (N18).

The category *meeting me where I am* also demonstrated that effective and comfortable doses required open, trusting and collaborative healthcare provider relationships.

*“Whether I was going down or going up* [in my dose]… *and even, more radical ideas of dropping doses…*[my doctor] *was pretty thorough in discussing it, and wasn’t trying to steer me in any specific* [direction]*.* [My doctor would] *make a suggestion but when I’d ask about the other aspect,* [my doctor] *would be able to give the information without it being biased or anything.”* (N05)

Sampling of participants who did not have the same involvement in dose-decisions provided further evidence for the connection between the main categories (i.e., opening up, being a part of care and receiving an individualized dose). When the prescribing physician was *“the one to suggest how much it* [my dose] *has to go up or down”* (N11), participants experienced difficulty finding a comfortable and effective dose. As this participant explained, *“I went through withdrawals for 2.5 months every morning…because I didn’t have my dose high enough…and when I would go to see* [my doctor]*, I would ask can you please up my dose and* [my doctor] *would say ‘no’…”* (N14). These alternative experiences occurred when participant’s unique needs and preferences were not met: *“*[My doctor] *doesn’t listen, and* [she/he] *just does what* [she/he] *wants to do…* [she/he] *still tries to push methadone on me…I tried to go up on my dose a couple of times in the past and* [my doctor] *wouldn’t let me, like* [she/he] *said ‘you gotta do this first … I want to do this, and try this’. And I’m trying to tell* [my doctor] *what I know works for me*” (N28).

Participants emphasized that holistic care be delivered in a manner that encouraged clients to *“have the responsibility to invest in* [our] *own lives* (N10). When healthcare providers took time to understand the evolving needs of their clients and were presented with information in an unbiased manner, they gained an increased sense of empowerment.

*“Today* [my doctor] *asked me if I wanted to do an anxiety test because it’s part of my treatment plan. So I went through and did a questionnaire and at the end,* [my doctor] *said ‘you are having a bit of extra anxiety from the looks of this. We do have, as you know, the counselor,* [and] *the psychiatrist. Do you think it might help you to do that? That’s always open for you’. And it wasn’t put to me like ‘you need this’. It was – ‘what do you think you might need? Here’s what’s available’. … I didn’t feel bad saying ‘actually, I don’t think I want to see a psychiatrist’, because* [my doctor] *still treated me exactly the same way.”* (N01)

### Discovering self-reported outcomes

The second research question explored the process of reaching outcomes that participants’ prioritized when initiating iOAT. This concept, ‘*discovering self-reported outcomes’,* revealed a person-specific process that unraveled from reduced street opioid use (Fig. 1).

*“I knew that eventually because of it* [iOAT]*, my life would get better obviously if I didn’t have to do those things. I would get healthier, uhm, I would stop going to jail, uhm my life would become stabilized, I could start to slowly build back relationships in my life with people that love me.”* (N23)

Reduced street opioid use was the most consistent initial iOAT goal and outcome described. This was primarily attributed to the daily prescription of the injectable medication. Yet, participant’s explanations of why and how this outcome was realized varied. Some participants credited this outcome to how well the medication suppressed cravings and withdrawal (e.g., *“I actually feel it and it gets me better”* (N28))*.* Others reflected that the accompanying financial or social costs of street-acquired opioid use were no longer worth the “risk”, because “*I risk my freedom every time I do it* [use street heroin]” (N25).

Narratives about “freedom” were also rooted in participants’ goals to disconnect from the *“constant struggle”* (N23) of daily street opioid use that was “*extremely anxiety inducing”* (N11). Participants attributed this struggle and anxiety to: *“living a day ahead because you don’t want to wake up sick”* (N30); *“spend*[ing] *our lives chasing the drug, or the money...there’s no time for anything else, but your addiction”* (N07); and “*having to steal sometimes in order to support that habit”* (N11). Being able to disconnect from this struggle and anxiety brought an increasing sense of *“stability”*, *“having a life”*, *normalcy”* and *“routine”*. These outcomes carried subjective meanings, including regular sleep, food in the cupboard, money left at the end of the month, being able to attend a movie or a concert, and reconnecting with family.

Within narratives of “stability”, participants defined positive changes to health functioning (e.g., *“weight gain”, “eating and sleeping better”*). Generally, participants felt they were taking better care of their health by prioritizing treatment for chronic conditions that had been neglected over the years (e.g., Hepatitis C treatment, dental and vision, medications and counseling for depression and anxiety). Such health outcomes were primarily discussed in relation to the delivery of holistic care that was part of this iOAT setting. Examples of how these outcomes arose further emphasized that building relationships (especially feeling supported, accepted, and understood) was fundamental to these outcomes. For instance, when the nurse practitioner took the extra time to go over a participant’s health history, this “*got me thinking more in terms of what do I need to just feel good for today, what can I do to make my future better, you know? Yeah, taking care of my pap tests, my breast exams and both my mom and grandma had breast cancer”* (N04).

## Discussion

The aim of this grounded theory study was to explore participants’ experiences with iOAT, as they broadly related to PCC. The findings suggested that therapeutic relationships were defined by mutual trust, respect and understanding. These relationships required time and space to open up and be a part of treatment decision-making. This process created opportunities for an individualized and holistic approach to iOAT.

Explanations regarding why it was necessary to open up reflected participants’ prior experiences of discrimination in the healthcare system. Examples of similar narratives are replete in qualitative research conducted in other settings, including MAT with oral opioids [39–41]. For instance, participants have previously described concerns about dose decisions being based on a “one size fits all approach” [42, 43], guided by the results of street heroin urinalysis [42, 44], and offered in inflexible spaces that perpetuate stigma and limit recovery [42, 44–48]. This extensive literature on dose decisions in the context of MAT with oral opioids supports understanding of why opening up was essential to developing positive therapeutic relationships.

These reports also provide an important background for considering how therapeutic relationships were built when some iOAT procedures (e.g., observed dosing; daily administration in one designated clinic; urine screens) could have posed similar power struggles. Yet, these features were not raised, even when this was probed for during data collection. Instead, participants explained that engaging in a consistently positive environment offset some of those challenges and allowed them to “let their guard down” over time. Further support for the role of time and environment in the process of opening up can be drawn from prior studies of people’s experiences in substance use treatment [49–58]. For example, clients receiving MAT with oral opioids have similarly emphasized “getting to know each other” as a gradual process rooted in mutual trust [49]. Likewise, healthcare providers have expressed that gaining the trust of clients is a “slow process” requiring multiple opportunities for meaningful engagement [52].

Upon establishing trust, participants felt safe to have “input into what we need…rather than being told what we need”. Despite the generally positive experiences described by participants, this was not a consistent finding. By sampling participants with alternative experiences, we gained further clarity regarding the connection between being a part of care and the therapeutic relationship. On the one hand, participants needed to establish mutual respect and trust for shared decision-making. However, feeling a part of treatment decisions also strengthened their relationships. These findings have been illuminated elsewhere [55, 56, 59]. For example, Ness et al. [55] found that participant experiences collaborating with practitioners required “not being judged”. In another study, Rance et al. [56] found that shared decision-making diminished perceptions of “adversarial relations” between clients and providers. These data suggest a benefit to client and provider collaboration, especially for aspects of treatment that are sensitive to individual preferences (e.g., dose decisions, choice of additional services) and that are within the healthcare providers’ ability to control [60, 61].

Opening up and being a part of care transpired in an individualized delivery of medications and services. The category “meeting me where I am” demonstrated that participants entered iOAT with unique needs and preferences. This provided an important framing for the second core concept, which revealed a person-specific unraveling of outcomes. The outcomes that participants identified were categorically consistent with prior iOAT qualitative studies [18, 19, 21] and the clinical trial outcome measures [62]. However, narratives in the present study demonstrated that therapeutic relationships and shared decision-making were required to reach the initial iOAT goal of reduced street opioid use. From here, further outcomes (i.e., reduced risk and stress, improved quality of life, health functioning) evolved in a manner that reflected variation in the complexity of participants’ opioid use history.

Therefore, findings support the importance of taking a broader bio-psycho-social perspective in MAT for opioid use disorder [40, 49, 61, 63]. This perspective would also need to consider individual variation in participant’s identification and prioritization of goals [64]. Some empirical research has been underway to develop [65, 66] or inform the development of patient-reported outcome measures in substance use treatment [67–70]. However, ongoing research is needed to determine how such tools reflect individually based goals and outcomes in MAT with oral and injectable opioids.

Beyond providing further understanding of the patient-centered aspects of iOAT, this study illustrates how PCC might be experienced in substance use treatment more broadly. This is particularly timely given increasing interest in the role of PCC for improving the quality of substance use treatment [29–33]. To our knowledge, few studies [33] have comprehensively explored the domains of PCC in substance use treatment according to existing conceptual frameworks [22–28, 32]. This study responds to this gap by demonstrating the fundamental role that therapeutic relationships play to the other dimensions.

Despite the novelty and timeliness of this research, there are potential limitations to the fullness of our findings. For instance, we sought to understand clients’ experiences with iOAT procedures that have been previously raised as barriers to MAT with oral opioids (e.g., observation and daily attendance). Despite efforts to sample and probe for varying experiences, participants expressed acceptance of these procedures in light of the current socio-political context. At the time of data collection, these clients were the only in North America to have access to iOAT. Therefore, as iOAT expands in North America, future studies should further explore the role of such procedures in providing patient-centered iOAT.

## Conclusions

To our knowledge, this is the first grounded theory study to explore participants’ experiences with the patient-centered aspects of iOAT. The core concept suggested that therapeutic relationships were fundamental to experiences of shared decision-making, individualized and holistic care. These findings fill important gaps regarding the attributes of care in iOAT and their relationship to participant’s self-reported outcomes. This research also provides a valuable example for further conceptualizing the process and impact of PCC in substance use treatment settings more broadly.

## Data Availability

The datasets generated and/or analysed during the current study are not publicly available due to the qualitative design that could potentially identify participants, but are available from the corresponding author on reasonable request.

## Abbreviations

iOAT: injectable opioid agonist treatment
MAT: Medication Assisted Treatment
PCC: Patient-centered care
